# Are Expectations the Missing Link between Life History Strategies and Psychopathology?

**DOI:** 10.3389/fpsyg.2018.00089

**Published:** 2018-02-06

**Authors:** Phillip S. Kavanagh, Bianca L. Kahl

**Affiliations:** ^1^ISN Psychology, Institute for Social Neuroscience, Heidelberg, VIC, Australia; ^2^The Florey Institute for Neuroscience and Mental Health, Heidelberg, VIC, Australia; ^3^School of Psychology, Social Work and Social Policy, University of South Australia, Adelaide, SA, Australia

**Keywords:** life history theory, psychopathology, evolutionary psychology, expectations, mismatch, predicative adaptive responses

## Abstract

Despite advances in knowledge and thinking about using life history theory to explain psychopathology there is still a missing link. That is, we all have a life history strategy, but not all of us develop mental health problems. We propose that the missing link is expectations – a mismatch between expected environmental conditions (including social) set by variations in life history strategies and the current environmental conditions. The mismatch hypothesis has been applied at the biological level in terms of health and disease and we believe that it can also be applied more broadly at the psychological level in terms of perceived expectations in the social environment and the resulting distress—psychopathology—that manifests when our expectations are not met.

## Introduction

Over the past 25 years there have been numerous developments and attempts to use evolutionary psychology to help explain the development of mental illness (see for example, Belsky et al., 1991; Ellis et al., 1999, 2017). More recently these attempts have made use of life history theory, conceptualizing mental illness in terms of behaviors associated with life history strategies, with traits associated with fast strategies initially identified as most reflective of mental health problems (e.g., Del Giudice, 2014, 2016; Hurst and Kavanagh, 2017).

All of these authors, and others, have made significant contributions to the thinking and understanding around the interplay between biology and the environment in shaping our developmental trajectory and in what clinicians refer to as *psychopathology*. We are going to argue that despite these advances in thinking, there is still a missing link. While we know that there are certain behaviors and biological markers associated with both fast and slow life history strategies, the question remains, “what causes some individuals to experience psychological distress, whereas others do not?” After all, having a fast life strategy is neither good nor bad from an evolutionary perspective—it is just adaptive^1^ (Kavanagh and Kahl, 2016). Is the missing link our expectations – a mismatch between our current life history strategy, the current environment, and a failure to appropriately adapt? Our rough blue print for what we can expect in our future environments is determined in part by both our parents’ early life experiences (i.e., epigenetic expression as result of their gene × environment interaction) and our own early life experiences (e.g., available nutrients in the womb, resource availability, and primary caregiver attachments during infancy and early childhood). The question is, what happens when our biological and cognitive expectations (our early developmental blueprint) are not consistent with what is happening in our current environment; including expectations from others?

We propose that some of the symptoms (cognitive, affective, and behavioral) of psychopathology are a result of a discrepancy between how we expect our environment to be, based on our current life history strategy, and our current environment. This expectancy mismatch results in a series of behaviors and cognitions—in an attempt to reduce the discrepancy—that are not adaptive in the current environment, ultimately manifesting in the form of a mental health disorder and/or triggering underlying genetic predispositions (i.e., diathesis-stress) for the emergence of disorders with known high genetic loadings (e.g., schizophrenia, bipolar disorder). Note, our proposition is not to discount the pathways proposed by Del Giudice (2014) from life history strategies to psychopathology (see the description of the model below), but rather plug what we consider a gap and explain the life history heterogeneity of disorders, such as depression and generalized anxiety disorder (Del Giudice, 2016), and offer an explanation for the large amount of comorbidity seen across mental health disorders.

### Life History Theory

At the strictly biological level, life history theory emerged out of the theory of evolution to help explain variations in species’ life cycles, maturation, reproduction, and social and sexual behavior (see Stearns, 1976, 1977). The theory used *r* and *K* to explain the balance (homeostasis) between the energy associated with reproduction, increases in population size, and the limiting factors from the environment (i.e., available resources). These terms were later extrapolated to be *r*-selected, or fast, and *K*-selected, or slow, strategies with specific reproductive and developmental behaviors associated with each strategy (Del Giudice et al., 2015). More recently the *r*-/*K*- categorical distinctions have been dropped in favor of conceptualizing life history strategies as being on a fast-slow continuum; especially when referring to humans, who are after all quintessentially a slow species by definition (Kavanagh and Kahl, 2016).

Our life history strategies develop as a result of our experienced environmental pressures. A faster life history strategy generally develops as a result of harsh and unpredictable environments, reflected in resource scarcity, inconsistent parental care and nurturance, and a higher risk of morbidity-mortality (Hill et al., 2008; Ellis et al., 2009). As a result of the environmental pressures, we have a propensity for quicker development and maturation (Nettle et al., 2013), as well as a tendency to discount future reward, in favor of short-term gains (Del Giudice et al., 2015). Conversely, a slower life history strategy generally develops in a resource abundant, predictable environment, associated with minimal risk to life. People with a slower life history strategy generally experience slower development with delayed maturation, and are more future orientated (Del Giudice et al., 2015). Although humans are a slow strategy species we do present with a large degree of within species variation (Kavanagh and Kahl, 2016). It is important to note that neither fast nor slow strategies are fundamentally better – the benefits of each strategy are contingent on the individual’s environment (Del Giudice and Ellis, 2014; Sung et al., 2016).

### Life History Model of Psychopathology

Life history theory has been used to explain developmental psychopathology with a life history model of psychopathology recently proposed by Del Giudice (2016). This model of psychopathology offers a classification system along the life history continuum, classifying disorders as fast, slow, or heterogeneous, dependent on the behaviors and traits that are characteristic of the disorder. The model proposes that the way in which a person configures their life history strategy may either increase or decrease their risk of developing certain mental disorders. That is, indicators of life history strategies can be reflective of symptoms of psychopathology and can possibly result in a set of difficulties (Del Giudice, 2016; Del Giudice and Ellis, 2016). Del Giudice (2014) and Del Giudice and Ellis (2014) propose four casual pathways from life history strategies to psychopathology: (1) adaptive life history traits could be considered symptoms of psychopathology; (2) life history traits could be expressed at a maladaptive level; (3) certain adaptive strategies may result in individually maladaptive outcomes; and (4) life history traits may increase susceptibility to dysfunction (Del Giudice, 2014, p. 269).

According to Del Giudice (2016) fast spectrum disorders are those that are characterized by traits such as risk-taking, sensation seeking, impulsivity, and disinhibition, low levels of agreeableness and conscientiousness, and promiscuous sexuality (e.g., substance use disorder, antisocial personality disorder). At the other end of the spectrum, slow spectrum disorders are characterized by risk aversion, extreme levels of agreeableness and conscientiousness, and restraint (e.g., obsessive compulsive personality disorder, social anxiety disorder; Del Giudice, 2016). The model suggests that psychopathology can occur at each end and along the continuum, and there has been some preliminary evidence in support of this model (see Hurst and Kavanagh, 2017); however, not all individuals experience psychopathology. So, while we agree with the classification of the mental disorders as per the life history model proposed by Del Giudice and colleagues, we contend that there may be a missing link – expectations. That is, we propose that a mismatch between our expectations set by our life history strategy and our current environment underlies and triggers the development of so called “psychopathology.” We believe that this helps to explain why only some people develop psychopathology and the heterogeneity in symptomology of more common mental health disorders, such as depression and anxiety noted in the clinical literature and as also described by Del Giudice (2016); alongside the common comorbidity seen across numbers of disorders.

## Mismatches Between Life History Strategy and the Environment

### Biological Mismatches

Issues can arise when our selected life history strategy no longer matches our environment. That is, predictive adaptive responses will only be adaptive if the environmental forecast is correct, otherwise these responses can be maladaptive and may result in poor physical health and disease (see Gluckman et al., 2005, 2007). Although we develop strategies in the pursuit of having an optimal adaption to our current environment, we also have the plasticity to adapt to environmental change; however, the flexibility and adaptive functionality of this plasticity is limited (Nettle et al., 2013). Adaption offers a trade-off, whereby we can either pursue an optimal survival under, and adaption to, a specific environment; or we can pursue an optimal adaption to changing environments and potential environmental extremes (Schmidt, 2011). Both of these abilities have their benefits, but also come at a cost. That is, should an organism actually develop an optimal adaption to a current environment, they would have a substantially increased chance of survival; however, at the cost of poor flexibility, which poses potential threat if the environment were to change.

Adaptive plasticity affects different mechanisms and operates on varying time scales from seconds to over 1000s of years. On one end of the continuum we have genome-based variation or natural selection to ensure diversity within a population (e.g., skin pigmentation, lactose tolerance; The 1000 Genomes Project Consortium, 2015) and by which future populations are provided with the opportunity for stable adaptive plasticity across generations (Ellegren and Sheldon, 2008). On the other end of the continuum, we have homeostatic mechanisms that allow for rapid and reversible adaptation, which assists in maintaining physiological and behavioral stability despite fluctuating environmental conditions (McEwen and Wingfield, 2003). For example, thermogenesis – the production of heat energy – is an imperative component of the homeostatic process which helps to maintain body temperature when we experience low environmental temperatures (Morrison and Madden, 2011). Somewhere in the middle of the adaption continuum we have epigenetic variation. Epigenetic variation is non-genomic adaption by which we can adapt within one generation or over a few generations through DNA methylation or histone acetylation (Fagiolini et al., 2009). Epigenetic variation causes no direct change to DNA sequences, but influences the expression or repression of genes. Epigenetic variation is highly adaptive as it increases an individual’s chance of survival as well as optimal fitness over their life course by preparing them for future environments (i.e., a predictive adaptive response). For example, there is increasing evidence the nutritional environment during fetal development can influence metabolism, growth, and brain development (Fagiolini et al., 2009), and variations in maternal care experiences during the immediate postnatal period are associated with changes in offspring’s hypothalamic-pituitary-adrenal activity, hippocampal plasticity, and neuroendocrine systems involved in reproduction (McGowan et al., 2009).

Despite the evolutionary advantage of having predictive adaptive responses in the form of a developed life history strategy, they also carry inherent risk of maladaptation if the expectations or the forecasts about an environment are incorrect (Nettle et al., 2013). These discrepancies between the expected environment and the actual environment are also referred to as a “mismatch” (Schmidt, 2011; Daskalakis et al., 2012). There are a vast number of research findings demonstrating the effect of incorrect environmental forecasts on development from biological, cognitive, and psychological perspectives.

A prime biological example can be demonstrated through the developmental origins of the health and disease model which argues that even before we are born we are making predictions about our future environments and how we can best adapt to these (Gluckman et al., 2007). Developing organisms have the ability to sense their metabolic environment and alter their physiological homeostatic set points in accordance with the environment they expect will exist post-partum (Gluckman et al., 2007; Gluckman and Hanson, 2008). If an organism receives signals during its development suggesting that available nutrients will be limited, the organism will adjust its developmental trajectory so that the matured individual has a metabolic homeostatic range that is better adapted for survival in an environment scarce in resources. In an effort to maximize reproductive fitness, individuals who anticipate nutritionally deficient environments have the tendency to prefer high-fat diets, invest in less muscle mass, binge, and are susceptible to visceral adipose stores when excess energy is available (Gluckman and Hanson, 2008). This adaptation has no adverse consequence if the individual’s environment is nutritionally limited; however, this is likely to lead to a mismatch if the individual’s forecast is incorrect, in that their actual environment is richer in nutrients than expected. Human lifestyle disease, including metabolic disease and obesity, often occurs when people live in environments beyond their environmental forecast and their consequential homeostatic range (Gluckman and Hanson, 2004, 2008). When an individual’s prediction is incorrect (i.e., their physiology does not match their developmental processes to the environment in which they will later live), the risk of disease is much higher. Conversely, when the individuals forecast environment and their actual environment match there is a much lower risk of disease (Gluckman et al., 2007; Champagne et al., 2009).

The increasing prevalence of obesity has been explained using the mismatch model. That is, we have unintentionally created a biological-environmental mismatch, as the human weight regulation system has not evolved fast enough to keep up with environmental change (Lee, 2009). The human weight regulation system evolved to help prevent weight loss, but is not overly effective in preventing weight gain. This could be due to the previous adaptions to ancestral periods of starvation – arguably starvation has a more devastating effect than over-indulging and could be an example of the smoke detector principle (see Nesse, 2005). In modern times, we have become increasingly sedentary, but we also have extremely easy access to processed food. Together, this results in increased energy intake and decreased energy expenditure. Although we have succeeded in creating productive and efficient work environments, we have inadvertently created a mismatch between our biology and environment. As a result there is a maladaptation of an otherwise efficient physiological mechanism, with substantial metabolic consequence (Lee, 2009).

### Cognitive Mismatches

The mismatch model has been applied to both human and other species’ cognitive functioning. For example, in rats, when deprived rats are exposed to high-stress levels they show enhanced learning over nurtured rats. Similarly, rats who are exposed to adverse postnatal environments display poor memory performance under non-stress conditions in adulthood; however, outperform their counterparts in a beneficial postnatal environment when tested in high-stress conditions (Champagne et al., 2008). These findings suggest that maternal factors determine optimal cognitive functioning in later life depending on the environmental demands, with highly nurtured rats and deprived rats showing enhanced cognitive function under contexts of low and high stress, respectively. A match between developmental and adult environment seems to promote successful adaption, while a mismatch between the two environments promotes disease and dysfunction (Champagne et al., 2008; Oomen et al., 2010).

Early life adversity is a known significant risk factor for later psychiatric problems (Laucht et al., 2000); however, not all people exposed to adversity develop a mental illness with some demonstrating a high degree of resilience. This resilience could be based on a favorable genetic predisposition, but it is also possible that the heightened resilience could be a result of an interaction between genetic predisposition and the match of the developmental environment and later adult environment. In research using rat models investigating the cumulative stress and mismatch hypotheses of psychopathology, findings supported a cumulative stress model, in that adult offspring who had been subjected to higher adversity were more susceptible to acute corticosterone than those who had experienced less early life stress and if there were a mismatch between the early-life environment and the later adult environment, there was an increased susceptibility to psychosis, supporting the mismatch hypothesis (Daskalakis et al., 2012).

Although much of the research in mental health and the mismatch hypothesis is currently reduced to experiments using rats, it is plausible to assume that these same principles apply in humans. The mismatch hypothesis has been supported in the context of explaining metabolic disease in humans, but research is required in the area of mental health. Along these lines Reser (2007) proposes that schizophrenia can be conceptualized as a predictive adaptive response to early life adversity. That is, schizophrenia represents an adaptive phenotype in response to early environmental events that signal later potential adversity that has potentially been around for at least 60,000 years (Reser, 2007). Reser (2007) draws links between the down regulation of metabolic activity in the hippocampal and frontal lobes associated with schizophrenia that are also associated with being adaptively hypometabolic in response to starvation – a predictive adaptive response to potential environmental adversity given the high energy costs of higher level cognition (for a full discussion see Reser, 2007). Taken together, the existing theoretical and empirical literature paints an interesting picture, where individuals’ adaptive phenotypic plasticity triggered as a result of environmental cues, can be further moderated by genetic variants. In turn, whether or not a programmed adaptation increases fitness or whether it is maladaptive is more so dependent on the accuracy of the individuals’ expectations or environmental forecast (Schmidt, 2011).

In humans, childhood adversity and family unpredictability have been implicated in the development of a childhood unpredictability schema that is associated with future discounting, impulsivity, and risk taking (Hill et al., 1997; Ross and Hill, 2002; Cabeza de Baca et al., 2016). These unpredictability schema may in turn shape our internalized working models of attachment and ultimately our perceptions of social support and how we perceive others will react to meeting our needs (Collins and Feeney, 2004). Indeed, recent research investigating the mediating roles of executive functioning and cognitive styles between life history strategies and socially undesirable personality traits (i.e., psychopathy) and behaviors (i.e., intimate partner violence and interpersonal aggression) indicates that there are a set of schema that are associated with variations in life history strategies (Figueredo et al., 2017). This suggests, that just as there is biological forecasting, there is the possibility of higher level cognitive forecasting (i.e., expectations) about the social environment and interactions with others. Figueredo et al. (2017) demonstrated—cross culturally—that a faster life history strategy is associated with an antagonistic social schema, which in turn is associated with higher rates of engaging in interpersonal aggression and intimate partner violence, and higher scores on a measure of psychopathy. Although this research did not investigate a mismatch, the indirect link through schema suggests individuals have set cognitive expectations about their environments. The mismatch here is between the behavioral and cognitive expression of a fast life strategy and what is expected within the larger society – what Del Giudice (2014) described as life history related traits being regarded as symptoms.

## Psychopathology

Extending the work examining biological and cognitive mismatches, we contend that psychopathology is a result of a mismatch between expectations of the environment and the current environment. People develop a reliable way of interacting with the environment that best enhances their chances of survival and reproduction – a life history strategy. Our life history strategies influence our cognitive flexibility and executive functioning (Mittal et al., 2015), our relationship processes in terms of selecting, pursuing, and maintaining a mating relationship (Gladden et al., 2008), our reproductive strategies (Kruger et al., 2013; Woodley of Menie et al., 2017), our eating behaviors (Hill et al., 2016),our attitudes (Wenner et al., 2013; Figueredo et al., 2017), our personality styles (Gladden et al., 2009; Jonason et al., 2012), and other behaviors such as risk taking and impulsivity (Hill et al., 2008; Griskevicius et al., 2011; Ellis et al., 2012). If our current environment no longer matches the one in which we developed or were expecting based on our early developmental experiences this is going to cause stress; especially if there is a failure to appropriately adapt (i.e., adaptive plasticity). For example, if someone with a slow strategy were placed in an environment that was unpredictable this would leave them with a decreased ability to cope based on their developed cognitive and emotional scripts, and ultimately psychopathology. They would not be psychologically prepared for such an environment and thus if faced with a threat to their life may develop anxiety, depression, post-traumatic stress disorder, or a dissociative disorders in reaction to that stressor. A loss of secure resources and relationships may also result in anxiety, depression, or an adjustment disorder. At the extreme end, excessive attempts to regain the continuity and/or security possibly associated with their developmental life history strategy may manifest in behaviors reflecting Cluster C (American Psychiatric Association, 2013) personality disorders (i.e., avoidant, dependent, and obsessive-compulsive), and eating disorders such as anorexia nervosa. Conversely, if someone with a fast life strategy encounters an environment that was predictable and required long-term investment over short-term gain there would also be a potential mismatch between their scripts and the environment. They would also not be psychologically or behaviorally prepared for such an environment where they may be faced with not getting their emotional or physical needs immediately met, which may cause initial anxiety and distress at the lack of immediate responsiveness, and in turn lead to perceived rejection causing depression or impulsive attempts at emotional regulation (i.e., self-harm, substance abuse). At the extreme end they may develop increases in goal directed behavior to get needs met (e.g., mania) or manifest behaviors associated with Cluster B (American Psychiatric Association, 2013) personality disorders (i.e., narcissistic, borderline, histrionic, antisocial). Although schizophrenia has been suggested as being a potential adaptive response to early life adversity (a fast life strategy) that could convey some adaptive advantages (e.g., Reser, 2007), others (e.g., Nettle and Clegg, 2006; Del Giudice et al., 2014) suggest that it is more likely the traits at the lower end of the spectrum (i.e., schizotypy), such as increases in creativity, artistic ability, and mating effort that convey the evolutionary advantage, and when those traits are at the extreme end associated with the sequelae of schizophrenia they become less adaptive. This suggests an ‘optimal range’ in which these traits match the environment and when there is mismatch, these traits become pathological in their expression.

The consequential stress from the mismatch between current life history strategy and current environment would likely result in a number of other biological and cognitive outcomes, including changes in hormone levels that are known to be associated with a number of different psychiatric conditions (see for example, McEwen and Wingfield, 2003; Mitropoulou et al., 2004; Pajer et al., 2012; Beck and Bredemeier, 2016; Kudinova et al., 2016; Zilioli et al., 2016). The poor adaptive flexibility in the face of mismatches might also help to explain some of the more pervasive disorders, such as some personality disorders (i.e., inflexibly attempting to use the same behavioral responses to the environment despite this not being the optimal strategy).

Although there is no direct empirical evidence to-date supporting our proposed missing link of a mismatch between life history strategy based expectations and current environment, as already cited above, there is a large amount of indirect evidence. Also anecdotally, the first author, who is a practicing clinical psychologist has observed that clients often appear to have unrealistic expectations of their environment, events, and other people. These unmet expectations often result in anxiety, distress, and dogmatic attempts to manipulate others and/or their environment to meet said expectations; and when they cannot, they engage in ruminative worry. Experiencing a lack of success at controlling others and their environments to maintain the status quo (i.e., life history set expectations) often results in low self-worth, irritability, and social withdrawal (i.e., symptoms of depression). These same clients appear to also vary across the life history strategy spectrum. Using psychotherapeutic techniques to help people develop flexibility in their expectations given their current environment (including expectations about various relationships), seems to help people reduce their levels of distress and symptoms of psychopathology. Often those that struggle the most appear to be the people with strong fixed beliefs (expectations) about situations and people.

## Future Directions

The obvious future direction is testing our assumptions about expectations, with the initial step being able to measure peoples’ expectations about their environments and determine if they are reflective of a selected life history strategy. We know that people who grow up in harsh and unpredictable environments tend to develop a faster strategy, but do they have a set of expectations about their environment? Does plasticity further moderate these expectations in that people who have favored flexibility over pursuing an optimal strategy fair better when faced with a change in environments (i.e., they more quickly adapt and thus do not experience ongoing distress)? An ultimate test would be to track people over time in terms of their early developmental environments, developed life history strategies, current environments, expectations, adaptive flexibility, and symptoms of various psychopathologies.

## Conclusion

While the proposition of expectations being the missing link still needs to be empirically tested, we believe that on the face of it, this is a worthwhile endeavor. All humans have a life history strategy, but not everyone develops psychopathology. Likewise, psychopathology is not an inevitable result of just having a fast strategy. The proposed mismatch between a person’s expectations based on their life history strategy and their current environment leads to a series of attempts to meet those expectations, which may present as symptoms of psychopathology. Our proposal provides at least one testable hypothesis – that is, do expectations moderate the associations between life history strategies and various mental health disorder presentations. The next step is to test our proposition and to determine whether our expectations are met, without falling into the oft leveled criticism of researchers trying to meet their expectations – confirmation bias.

## Author Contributions

PK and BK equally contributed to the writing and intellectual development of the paper.

## Conflict of Interest Statement

The authors declare that the research was conducted in the absence of any commercial or financial relationships that could be construed as a potential conflict of interest. The reviewer BB and handling Editor declared their shared affiliation.

## Footnotes

1 There is often a discrepancy between the terminology used by evolutionary psychologists and clinicians (i.e., clinical psychologists, psychiatrists) with regard to *adaptive*. Clinicians will tend to use adaptive as synonymous with *appropriate* or *socially desirable*; whereas, for evolutionary psychologists the term adaptive, merely means that the trait or behavior conveys some evolutionary advantage. So, to be consistent with others writing in this area (e.g., Ellis and Del Giudice, 2014; Hurst and Kavanagh, 2017), the term adaptive will mean a behavior or a trait that conveys a fitness maximizing strategy.
